# Research Advances in the Synthesis, Metabolism, and Function of Chlorogenic Acid

**DOI:** 10.3390/foods14111914

**Published:** 2025-05-28

**Authors:** Yuxin He, Shengming Mao, Yingying Zhao, Jing Yang

**Affiliations:** Key Laboratory of Quality and Safety Control for Subtropical Fruit and Vegetable, Ministry of Agriculture and Rural Affairs, Collaborative Innovation Center for Efficient and Green Production of Agriculture in Mountainous Areas of Zhejiang Province, College of Horticulture Science, Zhejiang A&F University, Hangzhou 311300, China; heyuxin2000@foxmail.com (Y.H.); maoshengming2@gmail.com (S.M.); yingyingzhao@stu.zafu.edu.cn (Y.Z.)

**Keywords:** chlorogenic acid, synthesis, structural genes, regulatory genes, functional applications

## Abstract

Chlorogenic acids (CGAs) are a group of important plant secondary metabolites produced in the phenylpropanoid metabolic pathway; they are formed via the conjugation of caffeic and quinic acids and are widely distributed across different plant species. Renowned for their multifunctional activities—including antioxidant, anti-inflammatory, antimicrobial, anticancer, antidiabetic, and anti-obesity properties—CGAs are versatile natural food additives with diverse industrial applications. This review summarizes five distinct CGA biosynthetic pathways, the structural and regulatory genes involved, and their key biological functions. The insights aim to facilitate a deeper understanding of CGA metabolism and streamline its exploitation in agriculture and human health.

## 1. Introduction

Chlorogenic acids (CGAs) are important plant secondary metabolites produced in the phenylpropanoid pathway. They are widely distributed in plants such as coffee, honeysuckle, *Eucommia ulmoides*, and burdock [1,2]. As conjugated phenolic compounds, CGAs have an aromatic ring as their backbone and undergo hydroxylation to form either hydroxycinnamic acid or caffeic acid. Upon reaction with quinic acid, caffeic acid forms the well-known CGA (also known as caffeoylquinic acid), with the molecular formula C_16_H_18_O_9_ [3,4,5,6].

To date, five synthetic pathways for CGAs have been identified, all of which begin with phenylalanine. Three of these are considered the primary pathways for CGA synthesis, and all involve the key enzyme hydroxycinnamoyl-CoA: quinate hydroxycinnamoyl transferase (HQT). HQT has been confirmed as one of the most important enzymes in CGA synthesis because it can catalyze the conversion of caffeoyl-CoA and quinic acid into CGAs [7,8].

CGAs perform many biological functions and confer benefits in plants, including enhancing plant resistance to adverse conditions [9], effectively defending against attacks by various insects and herbivores [10], improving cold tolerance in citrus fruits [11], and exerting antifungal effects on fruits [12]. CGAs also have important applications in the food industry. For example, they can be used as fruit preservatives to promote wound healing [13] and delay decay [12]. Additionally, due to their widespread presence in human food, CGAs are recommended as natural food additives and dietary supplements [14,15].

Beyond their roles in plants and food systems, CGAs offer significant antioxidant properties and multifaceted pharmacological benefits for human health. Emerging from numerous biological experiments, CGAs demonstrate remarkable activities across multiple domains, including antioxidant [16], hypoglycaemic [17], hypolipidaemic [14], anticancer [18], antitumor [19], and neuroprotective [20] effects. Their therapeutic profile further extends to antibacterial and anti-inflammatory capabilities. For example, CGAs have been shown to inhibit the growth of pathogens such as *Staphylococcus aureus* [21], *Klebsiella pneumoniae* [22], and *Candida albicans* [21]. By modulating key signaling pathways, specifically the mitogen-activated protein kinase (MAPK) and nuclear factor κB (NF-κB) pathways, CGAs suppress the production and expression of critical inflammatory cytokines, thereby alleviating conditions like intestinal injury [23]. In the nervous system, CGAs play a pivotal neuroprotective role, mitigating neuronal damage and reducing the risk of neurological disorders such as Alzheimer’s disease (AD) [24], Parkinson’s disease (PD) [20], and intracerebral hemorrhage (ICH) [25]. Notably, CGAs’ utility extends to metabolic health. By enhancing glucose and lipid metabolism, they actively prevent and alleviate obesity, combat diabetes, and lower blood lipid levels, positioning CGAs as a promising agent in the management of metabolic syndromes [17].

Leveraging their multifaceted pharmacological effects, CGAs have emerged as versatile agents in medical applications. By fostering intestinal homeostasis and modulating gut microbiota, CGAs show promise in managing metabolic syndrome and related gastrointestinal disorders [26]. Their incorporation into antibacterial and anti-inflammatory therapeutics further underscores their clinical utility [27], while preclinical studies hint at their potential as adjuvants in cancer treatment [28]. Such diverse applications have positioned CGAs as a focal point in contemporary biomedical research, attracting substantial interdisciplinary interest.

Over the past few years, researchers have explored CGAs in plants from diverse perspectives. Five distinct biosynthetic pathways and key enzyme genes, including *PAL* (*phenylalanine ammonia-lyase*), *C4H* (*cinnamate 4-hydroxylase*), *4CL* (*4-coumarate:CoA ligase*), and *HQT* (*hydroxycinnamoyl-CoA quinate hydroxycinnamoyl transferase*), have been characterized. Additionally, the regulatory roles of transcription factors such as MYB, WRKY, ERF, and bHLH in modulating these enzymes have been elucidated. This review synthesizes recent advancements in CGA research, encompassing its biosynthesis, metabolism, functional characterization, and bioactivity, alongside foundational studies from prior decades. By integrating insights from plant biology, pharmacology, and metabolic engineering, we provide a comprehensive overview of CGA’s structural diversity, biosynthetic pathways, transcriptional regulation, and translational applications. The objective is to establish a framework for optimizing CGA production in plants and harnessing its therapeutic potential across human health, food science, and agricultural systems.

## 2. Classification and Distribution

### 2.1. Types of Chlorogenic Acids

CGAs are formed through esterification reactions between trans-cinnamic acids (e.g., caffeic acid, coumaric acid, ferulic acid) and quinic acid. The primary members of the CGA family include caffeoylquinic acids (CQA), feruloylquinic acids (FQA), *p*-coumaroylquinic acids (*p*-CoQA), etc. (Table 1) [29,30].

CQA is a phenolic acid derived from caffeic acid and quinic acid, categorized into monocaffeoylquinic acids (monoCQA), dicaffeoylquinic acids (diCQA), tricaffeoylquinic acids (triCQA), and their derivatives based on the number and position of caffeoyl groups on the quinic acid core. The term ‘CGA’ usually refers to 5-*O*-caffeoylquinic acid (5-CQA), which is the most common form found in plants and belongs to the monoCQA subclass. Several isomeric forms of 5-CQA exist, including pseudochlorogenic acid (1-CQA, 1-*O*-caffeoylquinic acid), neochlorogenic acid (3-CQA, 3-*O*-caffeoylquinic acid), and cryptochlorogenic acid (4-CQA, 4-*O*-caffeoylquinic acid), which are differentiated by the substitution patterns of caffeoyl groups on the quinic acid moiety [29,31]. Naturally occurring CGA derivatives include 3-*O*-caffeoylquinic acid methyl ester (CAM) and 1,5-*O*-dicaffeoyl-3-*O*-[4-malic acid methyl ester]-quinic acid (MCQA) [32,33].

**Table 1:** Types of chlorogenic acids in plants (adopted from [6,29,30,34]).

Type	Classification	Name	Name
Caffeoylquinic acid (CQA)	Monocaffeoylquinic acid (monoCQA)	1-*O*-caffeoylquinic acid (1-CQA)	Pseudo chlorogenic acid
		3-*O*-caffeoylquinic acid (3-CQA)	New chlorogenic acid
		4-*O*-caffeoylquinic acid (4-CQA)	Cryptochlorogenic acid
		5-*O*-caffeoylquinic acid (5-CQA)	Chlorogenic acid
	Dicaffeoylquinic acid (diCQA)	1,3-dicaffeoylquinic acid	Cynarin
		1,5-dicaffeoylquinic acid	
		3,5-dicaffeoylquinic acid	Isochlorogenic acid A
		3,4-dicaffeoylquinic acid	Isochlorogenic acid B
		4,5-dicaffeoylquinic acid	Isochlorogenic acid C
	Tricaffeoylquinic acid (triCQA)	1,3,5-tricaffeoylquinic acid	
		3,4,5-tricaffeoylquinic acid	
Feruloylquinic acid (FQA)	3-Feruloylquinic acid		
	4-Feruloylquinic acid		
	5-Feruloylquinic acid		
*p*-Coumaroylquinic acid (*p*-CoQA)	3-*p*-Coumarinic acid		
	4-*p*-Coumarinic acid		
	5-*p*-Coumarinic acid		

FQA is synthesized through the esterification of ferulic acid and quinic acid. Its most prevalent isomer, 5-*O*-feruloylquinic acid, has the molecular formula C_17_H_20_O_9_.

*p*-CoQA forms via the reaction between *p*-coumaric acid and quinic acid, with 4-*O*-*p*-coumaroylquinic acid (C_16_H_18_O_8_) as the predominant isomer. Both compounds are widely distributed in various plants and exhibit antioxidant activity [6]. Within the CGA biosynthetic pathway, *p*-CoQA can be converted into CGA through the sequential action of hydroxycinnamoyl-CoA shikimate/quinate hydroxycinnamoyl transferase (HCT) and coumarate 3-hydroxylase (C3’H) [35].

### 2.2. Distribution of CGA in Plants

Studies have revealed significant variations in the primary composition and structure of CGA across different crops and species [3]. For instance, 5-CQA serves as the dominant CGA component in coffee and *Solanaceae* crops [3,14,30], while Schütz et al. [36] identified all four monoCQA isomers and six diCQA isomers in artichoke (*Cynara scolymus* L.). Among these, 1,5-di-*O*-caffeoylquinic acid exhibited the highest concentration (3.890 mg/g) in artichoke heads and pomace, whereas 1,3-di-*O*-caffeoylquinic acid became the major isomer in artichoke juice, followed by 5-CQA [36]. In a separate analysis, Sultana et al. [21] characterized phenolic compounds in sweet potato leaves and detected caffeic acid, CGA, and several isomers (e.g., ChA, 3,5-diCQA, 3,4-diCQA), with 3,5-diCQA reaching the highest levels (9.91–21.80 mg/g).

Beyond compositional and structural differences, the accumulation of CGA exhibits striking variability across plant species and tissues. For instance, Rosa-Martínez et al. [4] examined the phenolic profiles of tomatoes, aubergines, and peppers grown under identical conditions and reported CGAs of 1.81 mg/kg in aubergines, 25.0 mg/kg in peppers, and 25.0 mg/kg in tomatoes [4]. In a parallel study, Bellumori et al. [37] analyzed six Andean potato varieties, revealing a wide range of concentrations of CGAs in pulp (0.02–2.02 mg/g) and peel (1.59–14.81 mg/g), with 5-CQA accounting for 74% of the total phenolic acids. These findings are consistent with those of Valiñas et al. [38], who found that potato peel contained significantly higher levels of CGA (an average of 1.036 mg/g), which was 1.2 to 5 times greater than the level found in the pulp (an average of 0.423 mg/g). Ilie et al. [39] detected exceptionally high concentrations of CGA (187.435 ± 1.96 mg/g) in Crataegus extract using UHPLC-MS. Lu et al. [40] identified high levels of CGA (3.09 ± 0.353 mg/g) and 3,5-dicaffeoylquinic acid (14.42 ± 0.616 mg/g) in the chrysanthemum cultivar ‘HangBaiJu’.

In summary, CGA (particularly 5-CQA) represents a pivotal phenolic acid with broad distribution across plant species. It accumulates at high levels in *Solanaceae* crops such as peppers and tomatoes, as well as in hawthorn and artichoke. Beyond edible plant tissues, non-consumable parts such as potato peels, artichoke pomace, and artichoke juice serve as significant CGA sources, underscoring the compound’s diverse botanical niches. The striking variability in CGA’s composition, structure, and abundance across plant species and tissues not only reflects the intricacy of plant secondary metabolism but also highlights its immense potential for applications in agriculture, food science, and medicine.

## 3. Synthesis of CGA

### 3.1. Synthesis Pathways

CGA is synthesized via the phenylpropanoid metabolic pathway, a fundamental route in plant secondary metabolism that underpins the production of diverse compounds in angiosperms. This pathway generates a broad spectrum of secondary metabolites, including anthocyanins, coumarins, lignans, and CGA, with the latter serving as a key soluble phenolic compound in human nutrition [2].

**Figure 1:**
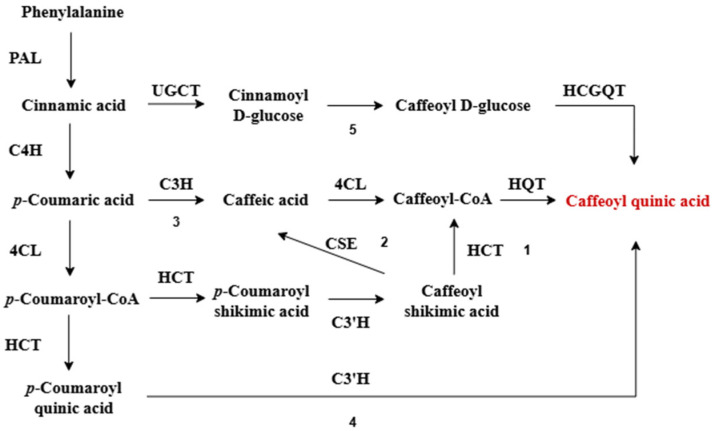
Biosynthetic pathways of chlorogenic acid in plants [34,41,42] (the numbers 1, 2, 3, 4, and 5 represent five different biosynthetic pathways of chlorogenic acid in plants, respectively).

CGA biosynthesis primarily occurs in the cytoplasm and chloroplasts of plant cells, with the final products transported to vesicles for storage. Five distinct biosynthetic pathways have been characterized, all converging on the phenylalanine ammonia-lyase pathway (Figure 1). [41,42]. The process begins with PAL-mediated deamination of L-phenylalanine, yielding trans-cinnamic acid—the central intermediate in phenylpropanoid metabolism [43]. In the first four pathways, trans-cinnamic acid is hydroxylated by cinnamate 4-hydroxylase (C4H) to form trans-4-coumaric acid (*p*-coumaric acid), which is then subsequentially modified by downstream enzymes to produce CGA [8,44].

In the first pathway, coumaric acid is activated by 4-coumarate/coenzyme A ligase (4CL) to form hydroxycinnamoyl-CoA [45]. Hydroxycinnamoyl-CoA shikimate/quinate hydroxycinnamoyl transferase (HCT) then catalyzes the formation of coumaroyl-shikimic acid, which is hydroxylated by coumarate 3-hydroxylase (C3′H) to yield caffeoyl shikimic acid. This intermediate is reconverted to caffeoyl-CoA via a second HCT-mediated reaction [46]. The final step, catalyzed by hydroxycinnamoyl-CoA: quinate hydroxycinnamoyl transferase (HQT), couples caffeoyl-CoA with quinic acid to produce CGA [10,35]. This pathway, prevalent in most plants, is regarded as the predominant route for CGA biosynthesis and has consequently been extensively studied [8].

In the second pathway, caffeoyl-shikimic acid is converted to caffeoyl-CoA through the combined action of caffeoyl shikimate esterase (CSE) and 4CL. HQT then catalyzes the transesterification of caffeoyl-CoA with quinic acid, yielding CGA [47]. Research has shown that CSE participates in lignin biosynthesis in *Arabidopsis thaliana* and influences CGA synthesis in diverse plant species. However, the role of this enzyme in mediating CGA’s involvement in plant lignin metabolism remains unclear and warrants further investigation [48,49].

In the third pathway, coumaric acid is hydroxylated by C3′H to form caffeic acid, which is then activated by 4CL to generate caffeoyl-CoA. Here, HQT catalyzes the final condensation step, coupling caffeoyl-CoA with quinic acid to yield CGA [34].

The fourth pathway diverges as hydroxycinnamoyl-CoA reacts with quinic acid via HCT to produce coumaroylquinic acid, which is subsequently hydroxylated by C3′H to form CGA [35].

In contrast, the fifth pathway is only found in a few plant species, such as sweet potato roots. In this pathway, cinnamate glucosyl transferase (UGCT) and quinate hydroxycinnamoyl transferase (HCGQT) act together to result in the formation of CGA [10,50].

Currently, the first three synthesis pathways are relatively common in plants, while the latter two are restricted to some plant species. Different plants exhibit diverse CGA synthesis pathways, with varying synthesis efficiencies and influencing factors. Consequently, more research is needed to explore the effects of different regulatory genes and other aspects of CGA synthesis.

### 3.2. Structural Genes

Both intrinsic factors and the external environment modulate the biosynthesis and metabolism of CGA. At the genetic level, these processes are governed by two primary classes of genes: structural and regulatory.

Structural genes directly encode enzymes critical for CGA biosynthesis, whereas regulatory genes orchestrate metabolic flux by controlling the expression of structural genes [51]. Key structural genes include *PAL*, *C4H*, *4CL*, *HCT*, *C3′H*, and *HQT*, with *UGCT* and *HCGQT* being uniquely associated with CGA biosynthesis in sweet potato (Table 2).

**Table 2:** Identified structural genes related to chlorogenic acid biosynthesis in plants.

Gene	Enzymes Encoded by Genes	Plant Source	Verification Method	Reference
*PAL*	Phenylalanine ammonia-lyase	*Nicotiana tabacum* *Dioscorea esculenta*	Gene expression Over-expression	Chen et al., 2023 [52] Liao et al., 2020 [53]
*C4H*	Cinnamate-4- hydroxylase	*Nicotiana tabacum* *Solanum tuberosum*	Gene expression Over-expression Transcription levels Expression patterns	Chen et al., 2023 [52] Valiñas et al., 2015 [38]
*4CL*	4-Coumarate:coenzyme A ligase	*Nicotiana tabacum*	Over-expression	Chen et al., 2023 [52]
*HCT*	Hydroxycinnamoyl-CoA shikimate/quinate hydroxycinnamoyl transferase	*Nicotiana tabacum* *Dioscorea esculenta* *Pyrus* *Citrus reticulata*	Gene expression Over-expression In vitro enzyme activity Transcriptome analysis Gene silencing	Chen et al., 2023 [52] D’Orso et al., 2023 [54] Hoffmann et al., 2003 [55] Liao et al., 2020 [53] Wen et al., 2022 [8] Xiao et al., 2024 [11]
*C3′H*	Coumarate 3-hydroxylas	*Nicotiana tabacum* *Solanum tuberosum* *Lonicera japonica* *Pyrus*	Over-expression In vitro enzyme activity Expression pattern Heterologous expression Transcriptome analysis	Chen et al., 2023 [52] Knollenberg et al., 2018 [46] Qi et al., 2017 [56] Pu et al., 2013 [57] Wen et al., 2022 [8]
*HQT*	Hydroxycinnamoyl-CoA:quinate hydroxycinnamoyl transferase	*Nicotiana tabacum* *Solanum lycopersicum* *Solanum tuberosum* *Dioscorea esculenta* *Lonicera japonica*	Gene expression Over-expression Gene silencing RNA interference Knockout	Niggeweg et al., 2004 [7] Payyavula et al., 2015 [35] Cardenas et al., 2021 [58] D’Orso et al., 2023 [54] Medison et al., 2023 [59]
*UGCT*	Cinnamate glucosyl transferase	*Dioscorea esculenta*	In vitro enzyme activity	Villegas et al., 1986 [50]
*HCGQT*	Quinate hydroxycinnamoyl transferase	*Dioscorea esculenta*	In vitro enzyme activity	Villegas et al., 1986 [50]

HQT, a member of the plant acyl-CoA-dependent BAHD superfamily [60], plays a pivotal role in CGA biosynthesis across the first three identified pathways [8]. Niggeweg et al. [7] first characterized the *HQT* gene in tobacco and tomato, demonstrating that overexpressing *HQT* in tomato significantly increased CGA accumulation, while silencing reduced it. These findings established HQT’s essential role in leaf CGA biosynthesis [7]. Similarly, Payyavula et al. [35] reported a >90% reduction in CGA levels and premature flowering in *HQT*-silenced potatoes. In *Nicotiana tabacum*, *HQT* silencing via RNA*i* decreased CGA content to 1% of wild-type levels without altering plant phenotype [58]. CRISPR-mediated gene editing in tomato further confirmed *HQT*’s dominance in CGA biosynthesis [54]. In sweet potato, overexpression of *IbHQT*-*g47130* doubled CGA accumulation, whereas silencing reduced it, implicating this homolog in CGA metabolism [59]. Collectively, these studies underscore *HQT* as a key regulator of CGA biosynthesis. The extensive characterization of *HQT* across diverse species provides a robust foundation for targeted metabolic engineering efforts.

Beyond HQT, C3′H and HCT, which are members of the plant cytochrome P450 (CYP450) superfamily, also contribute to CGA biosynthesis. Wen et al. [8] identified C3′H and HCT as key rate-limiting enzymes in CGA’s downstream biosynthetic pathways through transcriptomic analyses of pear fruits at different developmental stages. Knollenberg et al. [46] cloned the potato *C3′H* homolog *StC3′H*, which shares 99% sequence identity with tomato *C3′H*. In vitro enzyme assays confirmed its ability to enhance CGA synthesis, and *StC3′H*-overexpressing plants exhibited increased CGA accumulation. Qi et al. [56] identified CYP450 family genes in *Lonicera japonica* and cloned *LjC3H* and *LjC4H* homologs, suggesting their involvement in CGA biosynthesis. Chen et al. [52] overexpressed *PAL*, *C4H*, *C3H*, *4CL*, and *HCT* in tobacco, demonstrating their collective role in CGA synthesis. Notably, *HCT*-overexpressing transgenic lines showed a 54–149% increase in CGA content compared to wild type, underscoring *HCT*’s promotional effect. Hoffmann et al. [55] further supported *HCT*’s role in CGA biosynthesis. However, Cardenas et al. [58] observed stunting, delayed flowering, and multi-stem phenotypes in *NtHCT*-silenced tobacco plants without significant CGA content changes, highlighting discrepancies that warrant further investigation into *HCT*’s precise function in CGA metabolism.

### 3.3. Regulated Genes

Transcription factors (TFs), regulatory protein molecules, influence CGA biosynthesis by modulating the transcriptional expression of structural genes in the CGA pathway. Key TF families, such as MYB, WRKY, ERF, and bHLH, have been implicated in regulating the phenylpropanoid metabolic pathway [61,62]. For instance, these families play pivotal roles in orchestrating CGA biosynthesis by activating or repressing genes encoding enzymes like PAL, C4H, and HQT. Extensive studies have characterized TF-mediated regulation of CGA synthesis and metabolism across plant species. Using molecular biology techniques, numerous TF genes involved in CGA biosynthesis have been identified and cloned, with representative examples summarized in Table 3.

Rommens et al. [63] identified a novel MYB transcription factor, StMtf1, in potato. Ectopic expression of the modified *StMtf1^M^* in potatoes activated the phenylpropanoid biosynthesis pathway, driving *HQT* overexpression. Transgenic tubers exhibited a fourfold increase in chlorogenic acid (CGA), cryptochlorogenic acid (CCA), and neochlorogenic acid (NCA) levels, rising from 0.43 to 1.83 mg/g. This confirms StMtf1 as a positive regulator of CGA biosynthesis. In a 2013 study across five potato genotypes, Payyavula et al. [64] characterized multiple MYB transcription factors and demonstrated that *StAN1* modulated phenylpropanoid levels beyond the anthocyanin pathway, particularly enhancing CGA accumulation. In tobacco leaves, *StAN1* treatment induced a >25-fold increase in PAL enzyme activity and upregulated *NtbHLH1* expression, suggesting a cooperative regulatory role for *StAN1* and *NtbHLH1* in CGA biosynthesis [64].

Ding et al. [51] heterologously expressed *AtMYB12* in tomato, demonstrating that this transcription factor directly targets and activates key genes in the CGA biosynthetic pathway (*PAL*, *C4H*, *4CL*, *C3’H*, *HCT*, and *HQT*), thus leading to upregulated expression in transgenic fruits. This intervention resulted in a 16-fold increase in phenylpropanoid accumulation, including dicaffeoylquinic acid, indicating *AtMYB12*’s role in promoting CGA synthesis. The paralogous genes *AtMYB11* and *AtMYB111* exhibit similar functions: their heterologous expression in tobacco upregulates *NtPAL* and enhances CGA levels compared to wild-type plants [65,66]. Tang et al. [67] identified LmMYB15, a R2R3 MYB transcription factor in *L. macranthoides*, which directly binds to the promoters of downstream targets such as *4CL*, *MYB3*, and *MYB4* to drive CGA accumulation. Luo et al. [62] conducted transcriptomic analyses across 16 sweet potato genotypes and identified *IbGLK1*, a GOLDEN2-LIKE (GLK) transcription factor from the MYB superfamily’s GARP subfamily, that activates promoters of CGA biosynthesis genes (*IbHCT*, *IbHQT*, *IbC4H*, *IbUGCT*) to enhance CGA production.

In addition to the MYB transcription factor family, ERF, WRKY, and bHLH families also modulate CGA biosynthesis and metabolism. He et al. [68] characterized *NtWIN1*, an AP2/ERF transcription factor-encoding gene in tobacco leaves, demonstrating that it targets and regulates key genes in the phenylpropanoid pathway. CGA content analysis revealed that *NtWIN1*-overexpressing tobacco leaves exhibited a 50.15% increase, while *ntwin1* knockout plants showed a 23.63% decrease, compared to wild type, confirming *NtWIN1*’s role in promoting CGA accumulation. Additionally, *NtERF4a* binds to the *NtPAL* promoter to activate its transcription, thereby enhancing CGA biosynthesis through *PAL* gene-dependent pathways [69].

Wang et al. [70,71] identified *NtWRKY33a* and *NtERF13a* from tobacco genomes, demonstrating their direct binding to the *NtHCT* promoter to activate transcription. *NtWRKY33a* redirects metabolic flux towards CGA synthesis, thereby inhibiting total polyphenol accumulation in tobacco [70]. Conversely, *NtERF13a* enhances tobacco tolerance to salt and drought stress by promoting CGA biosynthesis and accumulation [71]. Another tobacco transcription factor, *NtWRKY41a*, also drives CGA production: *NtWRKY41a*-overexpressing lines exhibit a 36.21–72.43% increase in CGA content compared to wild type, while knockout lines show a ~60% reduction [72].

Using weighted gene co-expression network analysis (WGCNA) and k-means clustering, researchers identified CmERF/PTI6 (AP2/ERF family) and CmCMD77 (MADS box family) transcription factors in ‘HangBaiJu’. These factors modulate the expression of CGA biosynthesis genes such as *CmPAL1/2*, *CmHCT*, and *CmHQT* by regulating a downstream MYB-bHLH complex comprising *CmMYB3* and *CmbHLH143*, thereby orchestrating CGA biosynthesis [40].

In cucumber, the bHLH transcription factor CsMYC2 was identified as trypsin-responsive, activating secondary metabolite synthesis via the MAPK pathway [73]. Specifically, CsMYC2 upregulates *CsPAL* expression to increase phenylpropanoid compounds, including CGA [73]. Conversely, the transcription factor CsWRKY24 exhibits an apparent negative correlation with CGA levels, though its regulatory mechanism remains to be elucidated [73].

**Table 3:** Identified chlorogenic acid biosynthesis-related regulatory genes in plants.

Transcription Factor	Genetic Family Members	Plant Source	Regulation Method	Verification Method	Reference
MYB	*StMtf1*	*Solanum tuberosum*	Positive regulation	Inducing overexpression of *the StHqt* gene	Rommens et al., 2008 [63]
	*StAN1*	*Solanum tuberosum*	Positive regulation	Promote the increase in PAL enzyme activity	Payyavula et al., 2013 [64]
	*ATMYB12*	*Arabidopsis thaliana*	Positive regulation	Activate and upregulate the expression of important CGA synthesis genes such as *SlPAL*, *SlC4H*, *Sl4CL*, *SlC3H*, *SlHCT*, and *SlHQT* in tomatoes	Ding et al., 2022 [51]
	*AtMYB11*	*Arabidopsis thaliana*	Positive regulation	Promote upregulation of *NtPAL* expression in tobacco	Pandey et al., 2015 [66]
	*AtMYB111*	*Arabidopsis thaliana*	Positive regulation	Promote upregulation of *NtPAL* expression in tobacco	Pandey et al., 2014 [65]
	*CmMYB3*	*C. morifolium*	Positive regulation	Directly regulate the expression of structural genes *CmPAL1/2*, *CmHQT*, and *CmHCT*	Lu et al., 2024 [40]
	*LmMYB15*	*L. macranthoides*	Positive regulation	*LmMYB15* may directly bind to the promoters of *4CL*, *MYB3*, and *MYB4*	Tang et al., 2021 [67]
	*IbGLK1*	*Ipomoea batatas*	Positive regulation	Combining and activating the *IbHCT*, *IbHQT*, *IbC4H*, and *IbUGCT* promoters	Luo et al., 2024 [62]
WRKY	*NtWRKY33a*	*Nicotiana tabacum*	Positive regulation	Binding to *the NtHCT* promoter and activating its transcription	Wang et al., 2023 [70]
	*NtWRKY41a*	*Nicotiana tabacum*	Positive regulation		Wang et al., 2022 [72]
ERF	*NtWIN1*	*Nicotiana tabacum*	Positive regulation	Indirectly acting on *Nt4CL*	He et al., 2024 [68]
	*NtERF4a*	*Nicotiana tabacum*	Positive regulation	Binding to the *NtPAL* promoter and activating its transcription	He et al., 2023 [69]
	*NtERF13a*	*Nicotiana tabacum*	Positive regulation	Binding to the *NtHCT* promoter and activating its transcription	Wang et al., 2023 [71]
	*CmERF/PTI6*	*C. morifolium*	Positive regulation	Regulating downstream *CmMYB3* and *CmbHLH143* to affect the expression of *CmPAL1/2*, *CmHQT*, *and CmHCT*	Lu et al., 2024 [40]
bHLH	*StbHLH1*	*Solanum tuberosum*	Positive regulation		Payyavula et al., 2013 [64]
	*CmbHLH143*	*C. morifolium*	Positive regulation	Directly regulate the expression of structural genes *CmPAL1/2*, *CmHQT*, and *CmHCT*	Lu et al., 2024 [40]
	*CsMYC2*	*Cucumis sativus*	Positive regulation	Regulating the expression of downstream *CsPAL*	Wang et al., 2023 [73]
MADS- box	*CmCMD77*	*C. morifolium*	Positive regulation	Regulating downstream *CmMYB3* and *CmbHLH143* to affect the expression of *CmPAL1/2*, *CmHQT*, and *CmHCT*	Lu et al., 2024 [40]

## 4. Function and Application of CGA

CGA, recognized for its antioxidant and anti-inflammatory properties, is widely distributed in human diets and finds applications across food, medicine, and healthcare sectors [22].

### 4.1. Antioxidant

CGA exhibits antioxidant properties validated across multiple studies. Vieira et al. [74] demonstrated that CGA and *p*-coumaric acid interact with ascorbic acid to protect low-density lipoproteins from ferryl myoglobin-mediated oxidative damage.

Lu et al. [16] established an oxidative damage cell model by treating bovine intestinal epithelial cells (BIECs-21) with 400 μM H_2_O_2_, which significantly increased malondialdehyde (MDA) levels. When oxidatively damaged BIECs-21 cells were treated with 10 µg/mL CGA, the compound reduced cellular MDA content, decreased reactive oxygen species (ROS) levels, enhanced superoxide dismutase and glutathione peroxidase activities, and suppressed pro-apoptotic factors expression. Collectively, these effects enhanced cellular antioxidant capacity and alleviated H_2_O_2_-induced oxidative injury and apoptosis.

Lai et al. [75] treated mycotoxin-containing porcine alveolar macrophages with varying concentrations of chlorogenic acid, isochlorogenic acid A, and neochlorogenic acid. Low-dose CGA treatments (16, 32, 64 µg/mL) significantly reduced mitochondrial and subcellular reactive ROS levels, though paradoxically decreasing the antioxidant enzyme GPX4. Among the tested compounds, 64 μg/mL isochlorogenic acid A exhibited the most potent activity, potentially due to its greater number of hydroxyl groups in the molecular structure compared to 3-CQA and 5-CQA isomers, enhancing antioxidant capacity. However, all three CGAs induced cytotoxicity at 128 μg/mL [75].

Aside from its well-documented antioxidant activity, CGA demonstrates pro-oxidant characteristics, with its dual effects modulated by metal ion concentration and type. For example, alkali metal salts of CGA display potent antioxidant activity, with the sodium salt presenting the strongest performance [76]. Conversely, exposure to excessive levels of CGA, such as chronic accumulation in tissues, may induce pro-oxidative reactions in the gastrointestinal tract and liver. Therefore, maintaining optimal CGA intake is crucial in order to harness its beneficial properties [77].

### 4.2. Antimetabolic Diseases

Metabolic disorders, prevalent in modern populations, pose significant health risks. CGA emerges as a key regulator of intestinal homeostasis, with documented benefits in mitigating gut dysbiosis. Dietary CGA undergoes partial hydrolysis in the small intestine, releasing quinic acid and caffeic acids for systemic circulation, while intact CGA reaches the colon for microbial degradation prior to absorption [78,79].

Mechanistic studies underscore CGA’s modulatory effects on gut microbiota. Song et al. [26] demonstrated that CGA remodels microbial composition and metabolites, alleviating endoplasmic reticulum stress to preserve intestinal barrier integrity. In high-fat diet models, Ye et al. [80] reported that oral CGA supplementation reduced adiposity, normalized gut microbiota, elevated short-chain fatty acid production, and mitigated glucose intolerance and endotoxemia. Rectal administration of CGA in rats further revealed its capacity to modulate *Bifidobacterium acidophilus* extracellular vesicles, increase glycine availability, and attenuate post-infectious irritable bowel syndrome via anti-inflammatory mechanisms [81].

Beyond its gastroprotective effects, CGA modulates glucose and lipid metabolism, offering therapeutic potential against metabolic disorders such as obesity and diabetes. Kumar et al. [14] demonstrated that CGA activates AMP-activated protein kinase (AMPK), inhibits 3-hydroxy-3-methylglutaryl-CoA reductase (HMGCR), and enhances carnitine palmitoyl transferase (CPT) activity, thereby promoting lipid oxidation, suppressing cholesterol synthesis, and reducing fat absorption in humans. Kong et al. [82] reported that CGA combined with caffeine synergistically regulates fat-metabolizing enzymes via the AMPK pathway, inhibits 3T3-L1 adipocyte differentiation, and attenuates adipogenesis.

In diabetic models, Zhou et al. [17] innovatively complexed myofibrillar protein with CGA (MP-CGA), which normalized hyperglycaemia and hyperlipidaemia in type 2 diabetes mellitus (T2DM) rats. Mechanistically, MP-CGA restored gut microbiota homeostasis by increasing probiotic abundance and suppressing pathogenic bacteria, thereby stabilizing glucose and lipid profiles. Martins et al. [83] identified caffeic acid derivatives in *Solanum betaceum* Cav leaf extracts with potent antioxidant activity and inhibitory effects against α-glucosidase (IC_50_ = 1.617 mg/mL) and human aldose reductase (IC_50_ = 0.236 mg/mL). Pimpley et al. [84] further showed that 5-CQA inhibits lipase-catalyzed triolein hydrolysis (IC_50_ = 3.15 mM), underscoring the broader metabolic impact of CGA-related compounds.

Collectively, these findings highlight CGA’s role in modulating glucose and lipid metabolism, underlying its therapeutic potential for diabetes and obesity. Such properties position CGA as a promising candidate for natural, safe, and effective nutritional supplements or pharmaceutical formulations. However, the precise molecular mechanisms governing its actions require further elucidation.

### 4.3. Antiinflammatory

Inflammation represents a complex immune cascade initiated by tissue injury or toxic stimuli, with chronic inflammation often underlying diseases characterized by redness, swelling, heat, pain, and dysfunction [27,85]. The use of plant-derived secondary metabolites for anti-inflammatory therapy has gained significant research attention due to their multifaceted advantages [85]. CGA, renowned for its potent anti-inflammatory, antibacterial, and antiviral activities, has emerged as a promising candidate for managing various inflammatory diseases [27].

Ling et al. [27] recently reviewed CGA’s therapeutic roles and mechanisms in inflammatory diseases, highlighting its ability to intervene in digestive, nervous, and respiratory system inflammations through in vivo and in vitro studies. CGA exerts anti-inflammatory effects by regulating key signaling pathways and modulating inflammatory cytokine expression, underscoring its broad-spectrum therapeutic potential.

Nuclear factor κB (NF-κB), a pivotal nuclear transcription factor, mediates cellular stress responses and contributes to the pathogenesis of inflammatory diseases when overactivated [86]. Zhao et al. [23] demonstrated that in chronically induced intestinal injury models, CGA treatment suppresses the inflammatory response by inhibiting the p38MAPK signaling cascade, which is intricately linked to the classical NF-κB pathway. Concurrently, CGA attenuates NF-κB activation, reducing the production of the pro-inflammatory cytokine TNF-α and alleviating intestinal damage and associated inflammatory pathologies.

Wang et al. [86] explored the interplay between resolvin D1 (RvD1), a bioactive unsaturated lipid mediator with potent anti-inflammatory properties, and CGA. Their findings revealed that CGA upregulates RvD1 expression, which in turn suppresses the NF-κB inflammatory signaling pathway. Concomitantly, this intervention reduces pro-inflammatory cytokines (IL-1β, IL-6, and TNF-α), thereby alleviating induced liver inflammation and tissue damage. These results underscore CGA’s hepatoprotective potential.

Shi et al. [85] established a non-alcoholic fatty liver disease (NAFLD) model via high-fat diet (HFD) feeding and demonstrated that CGA mitigates HFD-induced chronic liver disease and steatosis. Mechanistically, CGA enhances intestinal *Bifidobacterium* abundance, reduces *Escherichia coli* colonization, restores gut microbiota balance, upregulates glucagon-like peptide-1 secretion, and improves insulin sensitivity, thus collectively ameliorating NAFLD pathogenesis. Taken together, these data position CGA as a promising therapeutic candidate for NAFLD and other chronic inflammatory disorders.

### 4.4. Antibacterial

In addition to its anti-inflammatory activities, CGA exhibits potent antimicrobial and antiviral properties. It combats diverse microorganisms, including bacteria, fungi, and viruses, by alleviating infections, with its antimicrobial efficacy linked to pro-/antioxidant mechanisms [76]. Mechanistically, CGA induces pathogen-specific K^+^ efflux, enhances cell membrane permeability, and causes membrane rupture, leading to leakage of cytoplasmic contents (e.g., nucleotides) and disruption of intracellular proteins, DNA, and RNA, ultimately driving pathogen death [76,87].

CGA demonstrates inhibitory effects against Gram-positive *Streptococcus pneumoniae* (minimum inhibitory concentration [MIC] = 20 μg/mL), Gram-negative *Shigella dysenteriae* (MIC = 20 μg/mL), Gram-positive *Bacillus subtilis* (MIC = 40 μg/mL), and the fungus *Candida albicans* (MIC = 80 μg/mL) [87,88,89]. Sultana et al. [21] reported that methanol extracts of sweet potato leaves, rich in CGA and its isomers, exhibited in vitro antibacterial activity against *Staphylococcus aureus*, *Streptococcus dysgalactiae*, *Klebsiella pneumoniae*, and *Candida albicans* [21,22].

Wang et al. [73] isolated eight CQAs from *Ilex pubescens* leaves, all of which suppressed influenza A virus infection. CQAs with higher caffeic acid substitutions displayed stronger anti-inflammatory activity, with 3,4,5-TCQA demonstrating the most potent antiviral effect against IAV. This compound attenuates inflammatory cytokine production via the Toll-like receptor signaling pathway, thereby exerting dual anti-inflammatory and antiviral effects [73,90].

### 4.5. Neuroprotective Effects

CGA provides neuroprotection by acting on the central nervous system, either by crossing the blood–brain barrier directly or by modulating systemic inflammation indirectly. Liu et al. [25] demonstrated that CGA mitigates intracerebral hemorrhage (ICH)-induced injury by suppressing the expression of extracellular matrix metalloproteinase inducer and matrix metalloproteinase-2/9, thereby reducing neuroinflammation, blood–brain barrier disruption, neuronal cell death, and brain damage. In an Alzheimer’s disease (AD) mouse model, Shi et al. [24] showed that CGA alleviates β-amyloid deposition via the SIRT1/PGC-1α/PPARγ signaling axis. This suppresses neuroinflammation, oxidative stress, and neuronal damage, while improving cognitive function. Combining CGA with moderate aerobic exercise has been shown to synergistically enhance these beneficial effects in AD models. In Parkinson’s disease (PD), CGA inhibits the excessive production of ROS through the AKT/Erk1/2 pathway, thereby reducing axonal damage and neuronal apoptosis in the 6-hydroxydopamine-induced PD mice. This intervention ameliorates oxidative stress, motor deficits, and behavioral abnormalities in the PD model [20].

Sleep is fundamental to human health, metabolic homeostasis, and physiological balance. Emerging evidence suggests that CGA may improve sleep quality. Hibi. [91] demonstrated that daily intake of caffeine-depleted coffee bean extracts enriched with CGA significantly improved subjective sleep quality, shortened sleep latency, and enhanced cognitive functions. These findings imply that CGA supports neural activity, potentially mediating its sleep-promoting effects. While plant-based beverages are generally associated with improved sleep and reduced cardiovascular risks, the specific contribution of CGA to these outcomes underscores its unique role in promoting sleep-related health benefits [92].

### 4.6. Anticancer

Research indicates that CGA’s pro-oxidant activity may selectively induce oxidative stress in cancer cells, thereby inhibiting tumor progression. Additionally, it exhibits anti-tumor effects by suppressing tumor cell proliferation, inducing apoptosis, and disrupting oncogenic signaling pathways [76,78]. CGA has demonstrated therapeutic potential against breast cancer, cholangiocarcinoma, pancreatic cancer, and colorectal cancer by modulating apoptotic pathways [18,93,94].

CGA has recently advanced to Phase I (NCT02728349, April 2016) and Phase II (NCT03758014, November 2018) clinical trials approved by China’s National Medical Products Administration (NMPA), positioning it as a promising cancer therapeutic with broad clinical potential [28]. Huang et al. [28] first demonstrated that CGA induces solid tumor differentiation while inhibiting tumor cell proliferation, migration, invasion, mitochondrial ATP production, and clonogenicity, thereby exerting potent anticancer effects. Mechanistically, CGA regulates apoptotic proteins via the Bax/Bcl-2 pathway, thereby suppressing the growth of 4T1 breast cancer cells and inducing apoptosis. This highlights its potential for targeted breast cancer treatment [18,95]. Liang et al. [93] identified aldehyde ketone reductase family 1 member B10 (AKR1B10) as a critical regulator of cholangiocarcinoma cell survival and tumor progression. CGA potently inhibits cholangiocarcinoma cell proliferation, migration, and invasion by targeting AKR1B10, while inducing apoptosis to combat the disease [93]. Recent studies further establish CGA as a cancer differentiation inducer that synergizes with anti-PD-1 antibodies to enhance antitumor efficacy, underscoring its translational potential in cancer immunotherapy [19].

### 4.7. Functions in Plant Production

Beyond its benefits for human health, CGA plays a pivotal role in plant biology. It enhances plant resilience against biotic (e.g., pathogen infections) and abiotic (e.g., drought and salinity) stresses, inhibits fungal proliferation, and inhibits postharvest fruit decay [9,10,11].

In fruit applications, CGA exhibits potent antifungal activity. It does this by promoting the accumulation of ROS and inducing oxidative stress in pathogens. This inhibits fungal growth and reproduction. For example, it is highly effective against *Penicillium expansum*, a common cherry tomato pathogen, thereby reducing the risk of infection and decay and improving storage quality [9]. Jiao et al. [12] demonstrated that exogenous CGA can delay postharvest decay in peach fruit infected with *P. expansum* by activating key genes and transcription factors in the salicylic acid signaling pathway, thereby enhancing defense-related enzyme activity. Similarly, CGA treatment in postharvest pears increases total phenolic and flavonoid levels, activates the phenylpropanoid pathway and boosts related enzyme activity, thus promoting wound healing effectively. These properties establish CGA as a promising natural fruit preservative for postharvest treatment, enhancing wound repair and delaying decay to maintain fruit quality [13]. In citrus, CGA plays a crucial role in cold tolerance as a key metabolite in the phenylpropanoid pathway. Silencing the *CiHCT2* gene, which is involved in CGA biosynthesis, significantly reduces CGA accumulation, disrupts ROS scavenging mechanisms, and impairs chilling tolerance. Conversely, exogenous CGA supplementation enhances cold stress resilience in citrus [11].

Apart from its role in fruit treatment and pathogen defense, CGA is an effective defense molecule against various insect and herbivore attacks on plants, and an inhibitor of insect growth [10]. For instance, Elliger et al. [96] discovered that CGA exhibits anti-nutritional properties against the tomato fruitworm.

### 4.8. Functions in Animal Production

CGA has emerged as a valuable bioactive compound in human health, leveraging its antioxidant, anti-inflammatory, and metabolic regulatory properties. Notably, these attributes extend to animal production, where CGA holds promise for enhancing growth performance, immune function, and product quality. This offers innovative strategies to improve animal product safety and sustainability.

In intensive livestock systems, animals face heightened oxidative stress that compromises growth. As a phenolic antioxidant, CGA mitigates this stress, positioning it as a promising feed additive. Liu et al. [97] demonstrated that dietary CGA supplementation in broilers activates the autophagy-mediated Nrf2-p62 pathway. This enhances the activity of endogenous antioxidant enzymes in the broiler gut, upregulates cytoprotective gene expression, reduces cell apoptosis, inhibits oxidative stress response, and ultimately promotes intestinal homeostasis and healthy growth in broilers.

In aged laying hens, Bi et al. [98] showed that 250 mg/kg dietary CGA improves egg quality parameters such as shell thickness, egg weight, and yolk color. This improvement is achieved by reducing serum malondialdehyde and upregulating mRNA levels of antioxidant enzymes. In aquaculture, Qu et al. [99] showed that CGA can act as an effective antiparasitic agent against white spot disease in freshwater fish caused by protozoan ciliates. It also inhibits the expression of pro-inflammatory cytokines in fish, thereby exerting an anti-inflammatory effect.

### 4.9. Fortification of Food with CGA

The increasing consumer demand for nutritionally dense, antioxidant-rich foods has driven substantial research into bioactive compounds like CGAs [100]. Abundant in common fruits, vegetables, and beverages, CGAs have garnered significant attention in recent studies owing to their multifaceted biological activities and potential health-promoting effects [101]. Fortification with CGAs not only enhances a food’s nutritional profile but also improves sensory attributes, extends shelf life, and boosts functional properties, positioning it as a versatile ingredient in functional food development [100].

Wang et al. [102] demonstrated that incorporating chlorogenic acid into autoclaved lotus starch-based foods effectively modifies their physicochemical and functional properties. Specifically, this intervention inhibits the formation of double-helical structures in lotus seed starch, reduces the viscoelasticity and thermal stability of the resulting gel, and significantly enhances its resistance to enzymatic digestion. Notably, dynamic in vitro digestion studies revealed that the lotus seed starch-CGA complex promotes the proliferation of probiotic strains—including *Bifidobacterium longum* subsp. *infantis*, *Lacticaseibacillus rhamnosus*, *Bifidobacterium adolescentis*, and *Lacticaseibacillus casei*—while increasing acetic acid production and enhancing CGA bioavailability [103]. These attributes collectively position CGA-fortified lotus seed starch as a promising ingredient for developing low-glycemic-index functional foods [102].

Song et al. [104] improved the utilization of curcumin by fabricating covalent β-lactoglobulin (LG)-dicaffeoylquinic acid (diCQA) complexes, achieving an encapsulation efficiency of 49–62%. This method improves the stability and bioaccessibility of curcumin in food systems, highlighting its potential for a wide range of applications in the food industry. In a related study, Pan et al. [105] found that conjugating rice protein hydrolysate (2.5%, w/v) with 0.025% CGA under alkaline conditions significantly enhanced the hydrolysate’s emulsification activity and physical and oxidative stability. This treatment effectively inhibits the oxidative deterioration of lipids during storage, providing a versatile approach to enhancing the stability, texture, flavor, and nutritional quality of dairy products (e.g., milk and ice cream) and coffee-based beverages.

## 5. Conclusions and Outlook

As a prominent plant phenolic acid, CGAs exhibit a wide array of biological activities, including antioxidant, antibacterial, anti-inflammatory, anticancer, hypoglycaemic, and hypolipidaemic properties. These attributes render CGAs highly promising for the development of functional foods and advancing medical applications, positioning them as a focal point in natural product research and pharmaceutical development [34].

While numerous studies demonstrate the beneficial effects of CGAs and their metabolites in humans, animals, and plants, the absence of controlled experiments limits the robustness of current data. Thus, more mechanistic investigations are needed to validate these effects and address potential confounding variables.

The identification of novel regulatory genes, transcription factors, and enzymes, along with the characterization of CGA biosynthesis and metabolic pathways, will deepen our understanding of CGA synthesis and facilitate the development of high-CGA plant varieties. Current knowledge regarding the genomic localization of CGA regulatory genes and their molecular mechanisms remains limited, as there are few published reports, which underscores the need for further investigation.

Map-based cloning approaches, including QTL-seq and genome-wide association analysis (GWAS), have proven effective in agricultural research. These methodologies enable rapid localization of CGA-related quantitative trait loci (QTL) in plants. By integrating molecular marker development and genotyping, fine mapping and map-based cloning of CGA-associated genes can be achieved, elucidating the molecular basis of CGA biosynthesis and accelerating functional breeding efforts.

## Data Availability

No new data were created or analyzed in this study. Data sharing is not applicable to this article.

## Abbreviations

The following abbreviations are used in this manuscript:

1-CQA	neochlorogenic acid
2-CQA	pseudochlorogenic acid
4CL	4-coumarate:CoA ligase
4-CQA	cryptochlorogenic acid (4-*O*-caffeoylquinic acid)
5-CQA	5-*O*-caffeoylquinic acid
AD	Alzheimer’s disease
AKR1B10	aldehyde ketone reductase family 1 member B10
AMP	Adenosine Monophosphate
bHLH	basic helix-loop-helix
BIECs-21	Bovine Intestinal Epithelial Cell Lines-21
C3′H	coumarate 3-hydroxylase
C4H	cinnamate 4-hydroxylase
CAM	3-*O*-caffeoylquinic acid methyl ester
CGA	chlorogenic acid
CQA	caffeoylquinic acid
CSE	caffeoyl shikimate esterase
CYP450	cytochrome P450 enzyme
diCQA	di-caffeoylquinic acid
ERF	Ethylene-Responsive Factor
FQA	feruloylquinic acid
GDSL	Glycine-Aspartic acid-Serine-Leucine
GLK	GOLDEN2-LIKE
GPX	Glutathione Peroxidase
GWAS	genome-wide association analysis
HCGQT	quinate hydroxycinnamoyl transferase
HCT	hydroxycinnamoyl-CoA shikimate/quinate hydroxycinnamoyltransferase
HFD	high-fat diet
HQT	hydroxycinnamoyl-CoA quinate hydroxycinnamoyl-transferase
ICH	intracerebral hemorrhage
MAPK	mitogen-activated protein kinase
MCQA	1,5-*O*-dicaffeoyl-3-*O*-[4-malic acid methyl ester]-quinic acid
MD	malondialdehyde
monoCQA	mono-caffeoylquinic acid
MYB	Myeloblastosis
NAFLD	non-alcoholic fatty liver disease
NF-κB	nuclear factor κB
PAL	phenylalanine ammonia-lyase
*p*-CoQA	*p*-coumaroylquinic acid
*p*-coumaric acid	trans-4-coumaric acid
PD	Parkinson’s disease
ROS	reactive oxygen species
RvD1	resolvin D1
SCPL	serine carboxypeptidase-like
T2DM	type 2 diabetes mellitus
TF	Transcription factor
TNF-α	Tumor Necrosis Factor-alpha
triCQA	tri-caffeoylquinic acid
UGCT	cinnamate glucosyl transferase
WRKY	WRKY domain-containing protein
